# 

**Published:** 1892-07

**Authors:** 


### CONTRIBUTORS TO VOL. VIII.

Albers, Dr. E. M., Victoria.
Bennett, Dr. T. J., Austin.
Beall, Dr. E. J., Fort Worth.
Baldinger, Dr. W. H., Galveston.
Cerna, Dr. David, Med. Dep. University Texas, Galveston.
Clopton, M. D., Prof. A. G., Med. Dep. University Texas, Galveston.
Crofford, M. D., Prof. T. J., Memphis, Tenn.
Christian, Dr. G. W., Burnett
Dyer, Dr. Isadore, Tulane University, New Orleans.
Daniel, Dr. F. E., Austin.
Houghton, Dr. R. E., Midland.
Hulen, Dr. Vard H., Galveston.
Jackson, Dr. E. B., Houston.
Keiller, M. D., Prof. Wm., Med. Dep. University Texas, Galveston.
Kennedy, Dr. James, San Antonio.
Lewright, Dr. J. O., Austin.
Lillard, Dr. Z. F., Tyler.
Magruder, Dr. F B., San Angelo.
Morris, Robert, Student, Charity Hospital N. O.
Morris, M. D., Prof. Seth M., Med. Dep. University Texas, Galveston.
Milliken, M. D., Prof. S. E., N. Y. Polyclinic, N. Y.
McVey, Dr. Bruce P., Ella.
Matthews, Dr. W. J., Austin.
Menger, Dr. R., San Antonio.
Osborn, Dr. T. C., Cleburne.
Pugh, Dr., T. J., Hearne.
Powell, Dr. W. M., Albane.
Price, Samuel J., Beaumont.
Rogers, Dr. W. N., Belton.
Swearingen, Dr. R. M., Austin.
Starnes, Dr. G. J., San Antonio.
Smith, Dr. M. M., Austin.
Smith, M. D., Prof. Allen J., Med. Dep. University Texas, Galveston.
Spohn, Dr. A. E., Corpus Christy.
Thompson, M. D., Prof. J. E., Med. Dep., Universitiy Texas, Galveston.
West, M. D., Prof. H. A., Med. Dep. University Texas, Galveston.
West, Dr. W. Beverly, Fort Worth.
Wingate, Dr. J. D., Comanche.
Wallace, Dr. D. R., Waco.
White, Dr. F. S., Austin.

### GENERAL INDEX VOL. VIII.

Contents July, 1892, to June, 1893, Inclusive.

Abdominal Work, Some Unusual Cases in Recent ....... 2
Acne, Treatment of ........................... 16
Antipyrine a Hemostatic ........................ 25, 65
Adjunct Professors Texas Medical College .............. 36
An Important Decision ............................ 73
Alvarenga Prize, The ............................ 77
Association of Southern Medical Colleges ............ 234, 243
Atagonism between Opium and Belladonna .............. 205
Austin District Medical Society .................. 224, 366, 416
Additional Accommodation for the Insane .............. 225
Antidote to Hydroganic Acid ...................... 362
Announcement: Dr. Hudson ........................ 367
Announcement and Programme T. S. M. A ............. 412
Arsenic vs. Cholera ............................. 141
"All that Glitters," etc. ......................... 146
A Surprising Decision ............................ 149
A Sigh of Relief ................................ 151
Advertiser, The Relation of, to Reader ............... 171
Another Iconoclast ............................... 182
A Timely Warning ............................... 109
Another Good Man gone Wrong ...................... 333
American Electro-Therapeutic Society ................. 366
Austin District Medical Society ..................... 518
Axis Traction Forceps ............................ 512
A Year on the Ambulance ......................... 507

Bill to Regulate the Practice ....................... 282
Bill, Public Health, in Congress ..................... 284
Biographical .................................... 266
Biography of Dr. D. Cerna ......................... 266
" Dr. J. F. Y. Paine ......................... 31
" Dr. H. A. West ........................... 33
" Dr. E. Randall ........................... 33
" Dr. Wm. Keiller ........................... 34
" Dr. A. G. Clopton ......................... 34
" Dr. Seth M. Morris ......................... 35
" Dr. J. E. Thompson ......................... 36
" Dr. T. D. Wooten ......................... 37
" Dr. A. J. Smith ........................... 136
Barker, Dr. W. L., Superintendent, etc. ................ 76
Bennett, Mrs., Death of ........................... 82
Beauties of the English ........................... 484

Book Notices—
Annual Universal Medical Sciences .......... 82
Anus, Rectum and Sigmoid Flexure, Diseases of .......... 376
Addresses and Essays, Lydstone .......... 197
Anatomy (Surgical) for Students .......... 201
Bright's Disease, A Treatise on .......... 201
Botany, A Manual of .......... 200
Bartholow's Hypodermic Medication .......... 377
Bandaging, Principles and Practice of .......... 115
Bryce's Pocket Practice .......... 377
Cholera in Europe and India .......... 157
Chest, Throat and Nasal Cavity, Diseases of the .......... 200
Chemistry, Manual of Practical, M. and P. .......... 379
Diseases of Children, A Text-Book of .......... 292
" of the Rectum, Anus, etc .......... 376
" " Lungs, Heart and Kidney .......... 246
" " Kidney and Bladder .......... 378
" " Eye .......... 246
" " Skin, A Practical Treatise on .......... 196
" " Stomach, Ewald .......... 197
" " Chest, Throat and Nasal Cavity .......... 200
" " Urinary Apparatus .......... 161
" " Skin, A Practical Manual of .......... 162
Diagnosis, Essentials of .......... 200
Fermentation, Infection and Immunity .......... 375, 528
Gould's Pocket Dictionary .......... 245
International Clinics .......... 291
International Medical Annual .......... 160, 485
Keating's New Pronouncing Dictionary .......... 83
Medical and Dental Register and Directory .......... 197
Manual of Practice of Medicine .......... 245
Manual of Practical M. and P. Chemistry .......... 379
Nervous Diseases, Text-Book of .......... 199
New Remedies, Notes on, Cerna .......... 374
Naphey's Therapeutics, Vol. I .......... 116
Nose and Throat, Bosworth .......... 159
Ophthalmology, Text-Book of .......... 377
Physical Diagnosis .......... 161
Physician Himself, The .......... 116
Pye's Surgical Handicraft .......... 158
Pocket Pharmacy, Aulde .......... 160
Surgical Anatomy for Students .......... 201
Surgery, American Text-Book of .......... 198, 293
Surgery, Text-Book on .......... 378
Tuberculosis of Bones and Joints .......... 198
Text-Book, American, of Diseases of Children .......... 292
Visiting List, Physicians .......... 245
Visiting List, Review's .......... 245
Water, Uses of in Modern Medicine .......... 162

Cerebral Syphilis........................................ 386
Cocaine in Urethral Surgery.............................. 398
Christmas Greeting....................................... 229
Cholera Disarmed: The Triumph of Genius.................. 140
Central Texas Medical Society............................ 150
Chrysalis and Butterfly.................................. 519
Clinical Reports, New Orleans.................. 217, 327, 507
" " St. Louis..................................358, 453
" " Dr. E. J. Beall...................................... 362
" Notes, Dr. E. B. Jackson............................. 55
Cystic Degeneration of Both Ovaries...................... 86
Compound Fracture of Femoral Bone........................ 98
Cholera.................................................. 108
Cerna, Dr. David, Biography of........................... 266
Cœliotomy, Recent Cases of............................... 295
Conservatism in Gynecology, a Plea for................... 440
Carbuncle, Culver's Treatment for........................ 70
Clopton, Prof. A. G., Biography of....................... 34
Comanche County Medical Society.......................... 15
Chloral in Treatment of Boils............................ 17
Cystoscope, the Value and Application of................. 20

Department of Therapeutics.............. 401, 447, 349, 502
" Diseases of Women...................................... 407
" Dermatology........................... 410, 324, 220, 505
" Practice of Medicine.................. 314, 352, 404, 450
" Obstetrics and Gynecology.............................. 318
" Surgery................................................ 359
" Chemistry and Toxicology............................... 360
Dermatology, Notes on................... 410, 220, 324, 357
Discussion on Dr. Cerna's paper.......................... 212
" Dr. West's paper....................................... 255
" Dr. Houghton's paper................................... 271
" Dr. Clopton's paper.................................... 245
Dr. Barker and the Pauper Insane......................... 110
"Dead Again"............................................. 114
Diphtheria, Treatment for................................ 18
Dr. West on Modesty...................................... 22
Dr. Bennett's Operation.................................. 511
Dr. McLaughlin's Book.................................... 521

Enteric or Typhoid Fever in the Young and the Old........ 133
Epoch (An), in Medical History........................... 230
Ergotine, a New Role for, Observations on................ 393
Eye-Glasses and Spectacles, the Wearing of, etc.......... 215
Erysipelas, Treatment for................................ 18
Eczema, Treatment of in French hospitals................. 72
Etiology of Puerperal Eclampsia.......................... 501

Fistula in Ano, Treatment for without Cutting............ 390
Femoral Bone, Compound Fracture of....................... 98
Fibro-Cystic Tumor, Laparotomy for....................... 269

Foreign Correspondence .......................... 67
Free-booter (The), Again .......................... 422

Gonorrhœal Vaginitis, Treatment for .......................... 71
Gonorrhœa, Treatment for .......................... 19
Gonorrhœal Infection in Women, the Present Status of .... 123
Galveston County Medical Society .......................... 223
Greeting, to Our Patrons .......................... 21
Gastro-Enteritis of Childhood and Infancy .......................... 167

"Halcyon-Reminiscenses" .......................... 372
Hydroganic Acid, Antidote to .......................... 362
Homeopathic Sanitation .......................... 143
"Hammock Method" for Plaster Jacket, Milliken .......... 359
Homeopaths (The), At It Again .......................... 367
Higher Education .......................... 281
Hepatic Colic, Receipt for .......................... 17
Hospital, the Sealy at Galveston .......................... 29

Inguinal Hernia, Radical Cure for .......................... 431
Iconoclast, An .......................... 182
In Trouble, Printer's Ink .......................... 381

John Sealey Training School for Nurses .......................... 7
Joint Session A. D. M. S. and the Tex. C. Med. Society .. 103

Kind Words .......................... 81
Keiller, Prof. Wm., Biography of .......................... 34
Kennedy Matter, The .......................... 12, 75
Kennedy, Dr. James, speaks .......................... 57

Local Societies in Relation to State Medical Association .. 73
Logan, Dr. Samuel, Death of .......................... 333
Lupus Erythematosus of the Face, etc .......................... 178
La Grippe, continued Form of Fever in .......................... 129
Legal Murder .......................... 421
Louisville Medical College .......................... 520

Malaria and Its Manifestations .......................... 339
Maggots in the Nose, Chloroform for .......................... 447
Multilocular Ovarian Cystic Tumor .......................... 347
Milliken, Dr. Sam. E., Hammock Jacket .......................... 359
Medico-Legal Society (Psychological Section in) .......... 366
Medical News and Miscellany—great many small paragraphs. 38, 76, 117, 153, 189, 237, 287, 238, 369, 424, 482, 522
Medical College, Southern, Association of .......................... 234, 243
Medical Congress, Pan-American .......................... 235
Mississippi Valley Medical Association .......................... 15
Morris, Prof. Seth M .......................... 35
Medical Department University of Texas .......................... 27, 332
Marine Hospital Service. Circular Letter .......................... 420

New Orleans Clinic. Reports by R. Morris .......................... 217, 327
Notes on Recent Meeting T. S. M. A .......................... 515

Open Letter (An) to Texas State Medical Assocation.... 9
Official Letter to Secretary West, T. S. M. A............. 12
Opium and Belladonna, Antagonism between............. 205
Our Rating, Special Notice to Advertisers....... ...... 166

Public Health Bill in Congress........................ 284
Principle vs. Expediency............................ 105
Paine, Prof. J. F. Y., M. D., Dean, Biography of......... 31
Prize, The Alvarenga.............................. 77
Pugh's (Dr.) Method in Tedious Labor................ 420
President's (the) Annual Recommendations, T. S. M. A.. 455
Phenacetine in Frequent Urination............ ....... 71
Publisher's Notes.................................
........ 379, 428, 485, 45, 83, 119, 162, 201, 247, 294, 337, 529
Paralysis Agitans, Lecture on ... ..................... 260
Plaster Jacket, Milliken's Modification of................ 359
Ptomaines from Pathological Urine .... ............... 366
Pan American Medical Congress....................... 517

Quarantine Conference............................. 371

Relation of the Advertiser to the Reader................ 171
" Sanitary, of Texas and Mexico.... ............. 252
" of Local Societies to the State Medical Association 73
Retroversion of the Uterus.......................... 91
"Retrench or Bankrupt." Letter from Sec'y West..... .. 9
Randall, Prof. Ed., M. D., Biography of............. ... 33
Radical Cure of Inguinal Hernia...................... 431
Roll of Honor Texas Medical College.................. 481

Soft Chancre, Treatment for......................... 17
Subcutaneous Ligation of Artery...................... 165
Sanitation, Homeopathic............................ 143
Special Notice.................................... 188
Southern Medical Colleges, Association of............234, 243
Sarcoma of the Ilium, Case of................. ....... 395
Solace for Solis, (a).............. .................. 112
Some Unusual Cases in Recent Abdominal Work......... 2
Superintendent Barker........................... .. 76
Suggestions for the Busy Doctor.......................17, 69
Summer Diarrhœa.......... ......................... 53
Smith, Prof. Allen J., M. D., Biography...............136, 153
Sentiment vs. Duty................................ 76
Suppurative Appendicitis........................... 495

Typhoid fever in the Very Old and the Very Young...... 133
" " Report of Eighteen Cases................ 249
Typhus Fever in Mexico.... ........................ 285
The Diploma-ed Danger ............................ 147
"The Kennedy Matter."..............................12, 57
Training School for Nurses, at Galveston............... 7
The Tramp Journal ................................ 186
Texas and Mexico, Sanitary Relations of .............. 232

Timely Warning, A .......................................... 109
Tri-State Medical Society, Ala, Ga. and Tenn........... 68
Texas State Med. Assn. 25th Annual Meeting ........... 455
" " " " Program and Announcement.... 455
" " " Minutes and Memoranda, 25th Meeting 412
" " " Association........................... 481
Texas Doctors, New.................................... 420
Texas Medical College. The...........................27, 232
Thompson, Prof. J. E., M. D., Biography of............ 36
" " " Letter from ....................... 67
Trachoma, Mechanical Treatment of..................... 49
Trachelorraphy ......................................... 489
The Propriety of Operative Interference in Tumors of the Breast..................................... 497

Urethritis, Non-Specific, Treatment for................. 383
Urethral Surgery, Cocaine in........................... 398
Uterus, Retroversion of ................................ 01
University of Texas, Medical Department of...........27, 623

Vesico-Vaginal fistula.................................. 407
Vegetable Pepsin? Is there a........................... 100

Wooten, Dr. T. D., Biography of....................... 37
We View with Alarm.................................... 148

Dr. J. D. Westervelt, Jr., Corpus Christi Light Guard—detailed as Hospital Sergeant.

Dr. F. R. Ramsdell, Lampasas, Surgeon to ——— Company, and acting as Senior Hospital Sergeant.

Dr. Haynes, Mobile, Texas, detailed as Hospital Sergeant.

The medical staff is not yet fully organized. We learn that Dr. Grizzard, Dr. Duffau and Dr. Dart, Regimental Surgeons, have resigned.

### AMERICAN PUBLIC HEALTH ASSOCIATION.

The next annual meeting of the American Public Health Association will be held at the City of Mexico, November 29th, 1892. Preparations on a grand scale are already being made by our local Committee of Arrangements to insure the scientific and social success of the meeting. The federal and State governments of the Republic of Mexico will be represented by special delegates, and the most prominent scientists and sanitarians of that country, as well as those of the Central and South American Republics, will take an active part in the meeting.

It is our earnest desire that the United States be not lacking in this respect, and that our government and citizens be honorably represented. We, therefore, solicit the co-operation of our federal and State authorities, respectfully requesting them to send delegates; we furthermore beg all our own worthy members to attend the meeting and prepare papers on whatever sanitary subject they may desire, these papers to be handed in to the Secretary on or before October 1st, in order that the translations (English into Spanish, and *vice versa*) be ready on time.

FELIX FORMENTO, M. D., President,
81 Esplanade Avenue, N. O.

DR. IRVING A. WATSON, Secretary, Concord, N. H.

